# Ulnar-Sided Wrist Pain: A Diagnostic Evaluation Guide From 30-Plus Years of Experience

**DOI:** 10.7759/cureus.53332

**Published:** 2024-01-31

**Authors:** Ather Mirza, Justin B Mirza, Luke C Zappia, Terence L Thomas

**Affiliations:** 1 Orthopedics, North Shore Surgi-Center, Smithtown, USA; 2 Orthopedics, Stony Brook University Hospital, Stony Brook, USA; 3 Orthopedics, St. Catherine of Sienna Hospital, Smithtown, USA; 4 Orthopedics, New York Institute of Technology College of Osteopathic Medicine, Old Westbury, USA; 5 Orthopedic Surgery, Thomas Jefferson University, Philadelphia, USA

**Keywords:** nuclear bone scan, wrist injuries, wrist, ulnar variance, arthrogram, foosh, lunotriquetral ligament, ulnar impaction syndrome, triangular fibrocartillage complex (tfcc), ulnar sided wrist pain

## Abstract

Introduction: While multiple ulnar-sided wrist pain (USWP) diagnostic evaluation guides have been presented, none have included original clinical data or statistical analysis. The purpose of this study is to provide a diagnostic evaluation guide derived from original clinical data and analysis to help clinicians arrive at a differential diagnosis for USWP.

Methods: Using a computer search of patients presenting with sprains, instability, and laxity of the wrist, 385 patient charts were identified. Patient demographics, mechanism of injury, subjective complaints, physical findings, and diagnostic test findings were reviewed. Statistical analysis was performed to determine sensitivity and specificity of diagnostic methods on their ability to identify lunotriquetral ligament tears, triangular fibrocartilage complex (TFCC) tears, and ulnar impaction syndrome. Diagnostic arthroscopy was used as the reference standard.

Results: Ninety-three patients, comprising 101 cases of USWP, were included in the study. The onset of injury was traumatic in 83 out of 101 cases with motor vehicle accidents (N=46) being the most common, followed by overuse (N=18), and a fall onto an outstretched hand (N=16). The ulnocarpal tenderness test exhibited sensitivity/specificity of 72%/33%; lunotriquetral ligament laxity test of 42%/62%; bone scan of 80%/33%; radiocarpal arthrogram of 90%/98% for TFCC tears and 50%/91% for lunotriquetral ligament tears; midcarpal arthrogram of 82%/86% for lunotriquetral ligament tears. The mean ulnar variance on standard posteroanterior view radiograph was 0.95 mm, increasing to 2.67 mm on gripping posteroanterior view.

Conclusion: Physicians should suspect a lunotriquetral ligament and/or TFCC tear with the acute onset of USWP following a loaded dorsiflexed mechanism of injury. Ulnocarpal tenderness tests and pre-operative ulnar variance measures are effective for increasing suspicion of USW pathology. Bone scans are helpful in diagnosing ulnar impaction syndrome in conjunction with radiographic findings. A combination of midcarpal arthrogram for lunotriquetral ligament tears and radiocarpal arthrogram for TFCC tears should be employed.

## Introduction

Ulnar-sided wrist pain (USWP) is a common, yet complex, injury faced by orthopedic surgeons. Due to the complex anatomy and biomechanics of the ulnar-sided wrist joint and the multitude of causes of USWP - fractures, dislocations, ligament tears, joint degeneration - these injuries can be difficult to diagnose and treat [1-3]. Over the years, as our understanding of the anatomy and kinematics of the wrist joint has progressed, so too has our understanding of ulnar-sided wrist pathology [2-6]. Despite the progress made, there is still controversy over the best method of diagnosis and treatment for these daunting injuries [7-11]. 

To date, at least five comprehensive review articles and one instructional course lecture have tried to integrate the full scope of USWP. Taleisnik [1] gave a thorough review of etiology of USWP by grouping causative factors into tendon, joint, and bone categories. Shin et al. [12] and Nakamura [13] also gave well-outlined diagnostic regimens. Sachar [8] compared the evidence for various treatments for three common causes of USWP: lunotriquetral ligament tears, triangular fibrocartilage complex (TFCC) tears, and ulnar impaction syndrome. The two most comprehensive reviews were published by Vezeridis et al. [14] and Watanabe et al. [7], providing detailed summaries of anatomy, physical exam, imaging studies, and treatment. However, none of these reviews have included original clinical data or statistical analysis of their own.

We present a retrospective chart review to evaluate the sensitivity and specificity of ulnocarpal tenderness tests, lunotriquetral ligament laxity tests (ballottement), bone scan, and arthrogram with respect to diagnosing intra-articular pathologies associated with USWP. For the purpose of this study, we chose to define USWP as lunotriquetral ligament tears, TFCC tears, and ulnar impaction syndrome. This study provides a diagnostic evaluation guide for USWP supported by original statistical evidence in conjunction with the lead author’s 30+ years of experience in clinical practice. 

## Materials and methods

Based on the retrospective nature of the de-identified chart review, this study was determined exempt from Institutional Review Board (IRB) review, with a waiver of informed consent in accordance with institutional protocol. Using ICD-9 codes 842.00, 842.09, 718.83 and 728.40 (Sprains, Instability, and Laxity of the Wrist) the principal investigator derived the study population via a computer search of patients who were seen for an office visit between January 1996 and November 2006. By these parameters, 385 patients were identified. The 385 patient charts were reviewed for diagnoses of USWP, and the inclusion criteria consisted of those patients who received an initial diagnosis of USWP. Patients were excluded from the study if they had complex regional pain syndrome, were lost to follow-up, under the age of 18, pregnant, or did not undergo diagnostic arthroscopy. The remaining 93 patients, comprising 101 cases of USWP, were included in the diagnostic study.

Clinical diagnostic measures

Physical examination of the patients consisted of ulnocarpal tenderness tests and lunotriquetral ligament laxity tests. Standardized techniques were utilized for each maneuver, respectively. In the ulnocarpal tenderness test, the wrist was placed in maximum ulnar deviation, followed by the application of axial load to the wrist and passive pronosupination of the forearm. Reproduction of pain was considered a positive test. Ulnocarpal tenderness tests were evaluated on their ability to identify lunotriquetral ligament, TFCC, and or ulnar impaction syndrome pathology. The lunotriquetral ligament laxity testing was performed with the physician grasping the lunate and triquetrum between both thumbs and index fingers. An alternating palmar and dorsal load was then repeatedly applied. Increased laxity and pain compared to the contralateral side was considered a positive test. Lunotriquetral ligament laxity tests were evaluated on their ability to diagnosis lunotriquetral ligament tears. Radiographic evaluation included wrist posteroanterior (PA), lateral, and oblique views, and a gripping PA view with the forearm neutral, the shoulder abducted to 90 degrees, and the elbow flexed at 90 degrees. Ulnar variance was measured, retrospectively, using the method of perpendiculars [15].

After initial physical examination and radiographs, patients were treated conservatively through splinting, occupational or hand therapy, and/or cortisone injection. Failure to respond to conservative treatment elicited further investigation through other diagnostic measures. Radiocarpal and midcarpal arthrograms were performed by the principal author. Radiocarpal arthrogram was retrospectively evaluated for its ability to diagnose TFCC tears and lunotriquetral ligament tears. Midcarpal arthrogram was retrospectively evaluated for its ability to diagnose lunotriquetral ligament tears. Bone scans were performed at other local facilities by various practitioners. During the time period of this study, patients were routinely advised diagnostic testing with a 3-phase bone scan followed by arthrograms of the wrist and on occasion, MRI. Bone scans were evaluated for detection of all relevant pathology, with special attention paid to potential ulnar impaction syndrome cases. Subsequent to a positive finding on one or more of the aforementioned diagnostic tests, eligible patients underwent diagnostic wrist arthroscopy which was then used as the gold standard for confirming ulnar sided wrist pathology.

Statistical analysis

The goal was to determine the efficacy of each diagnostic test: ulnocarpal tenderness, lunotriquetral ligament laxity, bone scan, midcarpal arthrogram, and radiocarpal arthrogram. This was achieved by using the findings from arthroscopy to perform sensitivity and specificity tests, followed by the calculation of 95% confidence intervals.

## Results

We report on 101 cases of USWP (53 female, 48 male), mean age: 40.8 years (range: 18-76 y). Dominant-sided injury comprised 64 of 101 cases. The onset of injury was traumatic in 83 cases. The most common mechanism of injury was motor vehicle accidents (MVA) (N=46), followed by overuse (N=18), fall onto an outstretched hand (FOOSH) (N=16), lifting (N=8), hyperextension (N=4), pulling (N=4), crush (N=3), and twisting (N=2). The mean duration of symptoms was 10.0 months (SD±16.3 m), with the pain described as moderate/severe in 81 cases. Fifty-six cases presented with concomitant complaints of the ipsilateral extremity. Thirty-six cases reported pain radiating into either the ulnar side of the forearm (17/36), ring and little fingers (13/36), or both (6/36). Clicking in the ulnar-sided wrist was present in 36 cases.

Pathology categories based on arthroscopy findings are summarized in Table 1. Diagnostic arthroscopy confirmed lunotriquetral ligament tears in 64 of 101 cases, TFCC tears in 42 of 101 cases, and ulnar impaction syndrome in 19 of 101 cases. The presence of two or more USWP pathologies was confirmed in 38 cases. Nineteen cases were absent of any lunotriquetral ligament tears, TFCC tears, and/or ulnar impaction syndrome on diagnostic arthroscopy. Additional findings on arthroscopy included scapholunate ligament tears (N=21) and synovitis (N=9). Of the 64 lunotriquetral ligament tears confirmed on arthroscopy, concomitant scapholunate ligament tears were confirmed in 13 cases (20%). Furthermore, signs and symptoms of median nerve compression were documented in 22 cases during retrospective chart review. Sensitivity and specificity results from the respective diagnostic tests are reported in Table 2.

**Table 1:** Pathology Categories Based on Arthroscopy Findings

Pathology Category	N=
Isolated Lunotriquetral Ligament (LTL) Tear	30
Isolated Triangular Fibrocartilage Complex (TFCC) Tear	9
Isolated Ulnar Impaction Syndrome (UIS)	5
Concomitant TFCC and LTL Tears	24
Concomitant UIS and TFCC Tear	4
Concomitant UIS and LTL Tear	5
Concomitant UIS, TFCC, and LTL Tears	5
No Ulnar-Sided Wrist Pain Pathology	19

**Table 2:** Sensitivity and Specificity of Diagnostic Tests a Arthroscopy was used as reference standard for a positive test. b Number of cases. c Percentage was taken from the number of total positives. d Percentage was taken from the number of total negatives. e The ballottement test was used to determine lunotriquetral ligament laxity. TFCC: Triangular Fibrocartilage Complex

Test ^a^	Test and Arthroscopy Performed # ^b^	Sensitivity (95% CI)	Specificity (95% CI)	False Positives # (%) ^c^	False Negatives # (%) ^d^
Ulnocarpal Tenderness	101	72% (63-82)	33% (12-55%)	12 (17)	23 (79)
Lunotriquetral Ligament Laxity ^e^	101	42% (30-54)	62% (47-78%)	14 (34)	37 (62)
Bone Scan	90	80% (72-89)	33% (0-87%)	2 (3)	17 (94)
Radiocarpal Arthrogram (TFCC)	94	90% (82-99)	98% (94-100%)	1 (3)	4 (7)
Radiocarpal Arthrogram (Lunotriquetral Ligament)	94	50% (38-62)	91% (81-100%)	3 (9)	31 (52)
Midcarpal Arthrogram (Lunotriquetral Ligament)	73	82% (70-93)	86% (74-99%)	4 (10)	8 (24)

Of the 18 confirmed ulnar impaction syndrome cases that underwent bone scan, 14 cases were positively identified. Moreover, of the 27 radiocarpal arthrograms that were false negative for lunotriquetral ligament tears and subsequently underwent midcarpal arthrogram, 21 of 27 were correctly identified on midcarpal arthrogram.

Radiographic evaluation of the wrist (N=101) revealed a mean ulnar variance of 0.95 mm (range, -4 to 5 mm) on standard PA view and a mean ulnar variance of 2.67 mm (range -3 to 6 mm) on gripping PA view. Of the 82 cases with confirmed USWP pathology, 49 cases presented with positive ulnar variance, 24 cases with neutral ulnar variance, and 9 cases with negative ulnar variance on standard PA view. On gripping PA view, 70 cases presented with positive ulnar variance and 12 cases presented with neutral ulnar variance.

## Discussion

A successful path to diagnosis starts with an extensive history and physical examination, focusing on the mechanism of injury. Beckenbaugh [16] stresses the importance of these steps to increase diagnostic suspicion. Furthermore, Reagen et al. [17] pointed out that lunotriquetral ligament sprains usually occurred from hyperextension and twisting of the wrist and that looking for these mechanisms facilitated the diagnosis of USWP. Our study lent evidence to this finding as the mechanism of injury in the majority (N=68) of cases fell into the categories of MVA, FOOSH, hyperextension, or twisting. We presume the ulnar-sided wrist is more vulnerable to forceful loading injuries because it is no longer well-equipped to handle these stresses.

Moreover, evolution has seen a progressive shift in the load-bearing characteristics of the wrist from ulnar to radial [3,18,19]. Today we know that the radius bears about 80% of the weight at the wrist joint, and the ulna only 20% [3]. This is further complicated by inconsistencies in the regression of the ulna and the ulnar variance that arises from this phenomenon. Previous literature has highlighted the inverse relationship between ulnar variance and its implications on TFCC thickness and ulnar-sided wrist injuries [20-22]. Gelberman et al. [23] reported the average ulnar variance in patients with normal wrists ranged from -0.7 mm in Swedish-born patients to as high as +0.7 mm in African-American patients. The mean ulnar variance in our diagnostic group was more positive than any of these distributions, at +0.95 mm. This disparity may lend credibility to the theory that impact from the ulnar styloid into the structures of the carpus leads to the pathologies underlying USWP. From these findings, we can postulate that an ulnar-positive variant combined with pronation and loading impact to the wrist creates a fertile ground for TFCC and/or lunotriquetral ligament injuries.

Previous studies have cited the value of bone scans and arthrography in diagnosing USWP [1,8,12,24-28]. Our findings support the research by Pin et al. [28], which found bone scan to be a reliable means of detecting pathology but serves little value in discriminating between pathologies. In the presented study, 80% (70/87) of positive bone scans were confirmed by arthroscopy. More specifically, our results suggest bone scan to be especially helpful in diagnosing ulnar impaction syndrome in conjunction with radiographic findings, as 78% (14/18) of confirmed ulnar impaction syndrome cases that underwent bone scan showed positive preoperative scans. 

In 1988, Zinberg et al. [29] determined that three separate arthrograms (radiocarpal, radioulnar, and midcarpal) were necessary for a complete evaluation. In a 102-patient study of TFCC tears, Shionoya et al. [30] reported a radiocarpal arthrogram sensitivity of 85% and specificity of 100%, similar to our findings of 90% and 98%, respectively. From these findings, we conclude that radiocarpal arthrograms are effective for confirming and ruling out TFCC tears. However, radiocarpal arthrograms exhibited low sensitivity (50%) and a high false negative rate (52%) for lunotriquetral ligament tear detection. In contrast, midcarpal arthrogram exhibited high sensitivity (82%) and a lower false negative rate (24%) for lunotriquetral ligament tears. Therefore, it is our belief that both radiocarpal and midcarpal arthrograms should be used in the diagnostic protocol for USWP.

This study has several limitations. Due to the relative age of this study and limited number of cases for analysis, we were limited in the diagnostic methods available for evaluation. Moreover, statistical analysis comparing single versus multiple pathology cases was not performed due to the limited number of single pathology cases. Furthermore, specific tear grades were not consistently reported across patient records, which may further complicate the diagnostic evaluation process. Additionally, median nerve compression was documented in 22 of 101 cases, as well as scapholunate ligament tears in 21 of 101 cases, and synovitis in nine cases. While not included in our scope for USWP, clinicians should be mindful of these pathologies coexisting when discerning a differential diagnosis. We believe this to be particularly relevant when a lunotriquetral ligament tear is suspected, evident by the coexistence of scapholunate ligament tear in 20% of confirmed lunotriquetral ligament tear cases. Lastly, due to the anatomical and biomechanical complexity of the ulnar-sided wrist joint, we chose a limited scope of study by defining ulnar impaction syndrome, TFCC tears, and lunotriquetral ligament tears as the diagnoses associated with USWP. A more expansive follow-up study including additional USWP pathologies, sub-categorical analysis based on specific tear grades, and the incorporation of MRI warrants future study. Nonetheless, our study provides a diagnostic evaluation guide derived from original clinical data and analysis to help clinicians navigate through the complexity of the ulnar-sided wrist joint and arrive at a differential diagnosis.

## Conclusions

Through retrospective analysis, our study provides a diagnostic evaluation guide to help clinicians navigate through the complexity of the ulnar-sided wrist joint and arrive at a differential diagnosis. Ulnocarpal tenderness tests, LTL laxity tests, and bone scans are effective tools in increasing USWP suspicion. Bone scans may also be particularly useful in diagnosing ulnar impaction syndrome in conjunction with radiographic findings. findings. Midcarpal arthrograms were effective in the diagnosis of LTL tears, while radiocarpal arthrograms were effective in the diagnosis TFCC tears. Furthermore, ulnar-positive variance was present in a majority of patients with USWP pathology. Lastly, additional findings on arthroscopy were mostly cases of medial nerve compression, scapholunate ligament tears, and synovitis. Although we did not specifically consider these diagnoses in the scope of our study, the physician should be mindful of these pathologies potentially coexisting when formulating a differential diagnosis for USWP. 
